# Development of a loop-mediated isothermal amplification assay for the rapid detection of Alongshan virus

**DOI:** 10.1099/jgv.0.002094

**Published:** 2025-05-08

**Authors:** Wenlong Huang, Meiyi Chen, Yiwen Wang, Li Li, Tianmin Niu, Xin Guo, Jiaxuan Wang, Kaifeng He, Zhengkai Wei, Quan Liu

**Affiliations:** 1 College of Animal Science and Technology, Foshan University, Foshan 528225, Guangdong Province, PR China; 2 College of Veterinary Medicine, Southwest University, Chongqing 400715, PR China; 3 Department of Infectious Diseases, Center for Pathogen Biology and Infectious Diseases, Key Laboratory of Organ Regeneration and Transplantation of the Ministry of Education, The First Hospital of Jilin University, Changchun 130122, Jilin Province, PR China; 4 Institute of Zoology, Guangdong Academy of Sciences, Guangzhou 510260, Guangdong Province, PR China

**Keywords:** Alongshan virus (ALSV), loop-mediated isothermal amplification technique (LAMP), PCR, rapid detection, visualization

## Abstract

Alongshan virus (ALSV) is a recently discovered tick-borne zoonotic virus. Currently, there is no rapid and accurate clinical method for ALSV detection. This study aimed to develop a loop-mediated isothermal amplification (LAMP) assay for precise ALSV infection detection. Specific primers were designed based on the S1 segment of the ALSV NE-TH4 strain’s genome (GenBank accession no. ON408067.1). The reaction time, temperature and concentration of the neutral red staining solution in the LAMP assay were optimized. Thorough evaluations of specificity, sensitivity and repeatability led to the development of a visually interpretable LAMP assay. The optimal amplification time was 50 min. The minimum detection limit for cDNA was as low as 0.005 pg μl^−1^, and sensitivity for standards was 1.68×10^3^ copies per μl, surpassing that of PCR and real-time PCR. No cross-reactivity was observed with Jingmen tick virus, Bole tick virus 4 and Beiji nairovirus. These results indicate that the LAMP assay is more sensitive and accurate than PCR and real-time PCR. The developed LAMP assay allows for on-site detection, reduces testing costs and provides rapid and accurate results. Thus, it lays a solid foundation for the prevention and control of emerging tick-borne ALSV.

## Data Availability

The three sets of primers for the LAMP assay were designed based on the S1 segment of ALSV NE-TH4 strain’s genome (GenBank accession no. ON408067.1). The data that support the findings of this study are available in figshare (https://doi.org/10.6084/m9.figshare.28588604.v3)

## Introduction

Alongshan virus (ALSV), a recently identified zoonotic tick-borne virus, belongs to the *Jingmenvirus* group within the *Flaviviridae* family. ALSV infection causes Alongshan fever, manifested by clinical symptoms such as headache, fever, depression, malaise and dizziness. Tick bites have been reported in most patients [1]. In China, *Ixodes persulcatus* is considered the primary vector for these emerging viruses [2,4]. Additionally, ALSV RNA and virus-specific antibodies have been detected in sheep and cattle in Hulunbeier, northeastern Inner Mongolia [5]. The genomic segments of ALSV are classified into four types, numbered 1 to 4. Segments 1 and 3 exhibit monocistronic characteristics, while segments 2 and 4 are bicistronic features [6]. Specifically, segment 1 encodes proteins associated with Flavivirus that encompass RNA-dependent RNA polymerase and methyltransferase motifs [7]. Since its initial discovery in northeastern China in 2019, ALSV has also been detected in *Ixodes ricinus* in Switzerland, Finland and France, as well as in *I. persulcatus* from Russia. This indicates a wider distribution of ALSV than initially expected and highlights its potential public health implications [8,12]. In the absence of effective vaccines and therapeutic drugs against ALSV infection, developing a reliable methodology for epidemiological investigations of this newly identified zoonotic virus is crucial.

Viral detection technologies, such as viral isolation, nucleic acid detection and ELISA, are extensively employed in the diagnosis and epidemiological investigation of viral infectious diseases [5,13, 14]. Among these, viral isolation by cell culture is the most direct and accurate diagnostic method, yet it is time-consuming and labour-intensive. Nucleic acid detection techniques, like PCR and real-time fluorescent quantitative PCR, offer high accuracy and reliability but require specific equipment and conditions [15]. ELISA is rapid and sensitive, but suffers from poor reproducibility and high testing costs, limiting its further development [16]. Loop-mediated isothermal amplification (LAMP), developed by Notomi in 2000 [17], involves designing four specific primers for six regions of the target gene and amplification at a constant temperature of 60–65 °C using *Bacillus stearothermophilus* (Bst) DNA polymerase. Detection results can be obtained within 15–60 min. The basic principle of LAMP is the dynamic equilibrium of DNA at approximately 65 °C, where four distinct primers are utilized by a strand-substituted DNA [18]. LAMP has been successfully applied for detecting RNA viruses including SARS-CoV-2 [19], avian influenza [20], Beiji nairovirus (BJNV) [21] and Human immunodeficiency virus (HIV) [22]. The present study aimed to establish a LAMP assay as a rapid detection method for ALSV diagnosis and investigation, providing valuable insights for epidemiological research.

## Methods

### Virus strains and clinical samples

The ALSV, Jingmen tick virus (JMTV), BJNV and Bole tick virus 4 (BLTV4) have previously been described in the literature [1,23,25]. The ALSV virus strains utilized in this study were obtained from virus cultures maintained in our laboratory. JMTV-, BJNV- and BLTV4-positive tick samples were initially detected using reverse transcription PCR (RT-PCR) and subsequently identified through sequencing.

### Primer design

The LAMP assay necessitates four primers: two outer primers (F3 and B3), a forward inner primer (FIP) and a backward inner primer (BIP) [26]. The three sets of primers for the LAMP assay, as well as the primers for the conventional PCR, were designed based on the S1 segment of ALSV strain NE-TH4 (GenBank accession no. ON408067.1). The primers for real-time PCR were designed according to the S3 segment of the genome. Primer design was conducted using Primer Explorer V5 online software (https://primerexplorer.eiken.co.jp/e/), in combination with Primer Premier 6 and Oligo 7 (Table 1). The primers, synthesized by Sangon Biotech (Shanghai), were purified by PAGE. PCR and real-time PCR were employed to validate and compare the accuracy and sensitivity of the LAMP method. The sequences and positions of the primer sets within the ALSV strain NE-TH4 genome S1 segment sequence are detailed in Fig. S1, available in the online version of this article.

**Table 1.** The primer sequences of LAMP, PCR and real-time PCR used in this study

	Name of the primer	Sequence details (5′−3′)	Prime length (bp)
LAMPPrimer set 1	F3-1	GACCTGTGGCTCAAACGG	18
	B3-1	TGTTGTAGACGGCATGGGT	19
	FIP-1	TCCGCGCCTGTTTATGGCAGGTGGGGAGACTGCGTAGA	38
	BIP-1	ACCTCGGGTCCTACTGGGACGGGTACCTGCCAACAAGC	38
LAMPPrimer set 2	F3-2	TGAACATGACCAGATCCGTG	20
	B3-2	GGTGTCGTTGAGGTCAGC	18
	FIP-2	TACAGCCACTCTGTACCCCACCTGGACAGAGCACCACTCG	40
	BIP-2	GGCTCCGAAGGAACAAGGAAGACACATGACTCCGCTCAAGC	41
LAMPPrimer set 3	F3-3	GCCTGAACCTCAAAGGCATA	20
	B3-3	GCCCTCGACTCGTAAGCT	18
	FIP-3	CTTCCGTCTGTCACCAGGGCGGCTGGATGATGGACACG	38
	BIP-3	ATGGCAGTAGGAAGGGGCTCTAGTCCTCTTCTCCAGTGCTT	41
PCRPrimer set	F	GGACGTCAACACCTTTAAGCA	21
	R	GGTAACAAAGTTGGTGATCGTG	22
real-time PCR	NS3-QF	GGCTAAACACATCAAACA	18
Primer set	NS3-QR	GCATCCAGGTCATAGTTA	18

LAMP, loop-mediated isothermal amplification; real-time PCR, real-time fluorescence quantitative polymerase chain reaction.

### Viral RNA extraction and preparation of standards

The viral RNA was extracted using the TIANamp RNA Kit [Tiangen Biochemical Technology (Beijing) Co., Ltd, China] and reverse transcribed into cDNA using the RevertAid First Strand cDNA Synthesis Kit (Thermo Fisher Scientific Co., Ltd, USA) according to the manufacturer’s instructions. The ALSV cDNA was used as a template to generate LAMP and PCR standards employing the F3-3/B3-3 and F/R methods. The reaction conditions included an initial denaturation step at 94 ℃ for 5 min, followed by 35 cycles of amplification consisting of 30 s at 94 ℃, 30 s at 60 ℃ (or alternatively, 55 ℃) and finally 72 ℃ for another 30 s. A final extension step was performed at 72 ℃ for a duration of 5 min. Subsequently, the resulting amplified products were cloned into the pMD18-T. After shaking the bacterial solution well, 100 µl was taken and coated on a screening plate containing Amp and placed face up for half an hour. After the bacterial solution was completely absorbed by the medium, the Petri dish was inverted and cultured at 37 ℃ for 12 h. Finally, the strains were selected on the Petri dish, the bacterial fluid obtained was sent for sequencing, and the results were compared with ALSV strains on NCBI. ALSV-LAMP standards were obtained by extracting plasmid.

### Establishment and optimization of the ALSV-LAMP

The ALSV-LAMP method was developed based on methods described in previous works [20]. In brief, the synthesized LAMP primer sets were diluted to a concentration of 10 µmol l^−1^ and subsequently subjected to screening for identification of suitable primers. This screening process involved employing the reaction system and procedure provided by Sangon Biotech’s 2×Lamp PCR Master Mix (Universal). The negative nucleic acid samples were derived from the same batch of ALSV-free tick samples obtained in 2019 that detected ALSV in this experiment. The primer set identified through this screening was utilized for testing negative nucleic acid samples from the same batch in which ALSV was detected. The initial experimental parameters were established based on the LAMP reaction system recommended by Sangon Biotech. The total LAMP reaction system was 25 µl that comprised 12.5 µl of 2×Lamp Master Mix, 0.8 µM of each FIP and BIP, 0.2 µM of each F3 and B3, 0.16 U µl^−1^ of Bst-DNA Polymerase, 2 µl of template DNA and 5 µl of ddH_2_O. The reaction procedure involved maintaining a temperature of 65 ℃ for a duration of 60 min, followed by a final step at 80 ℃ for 10 min. To visualize the LAMP reaction, Neutral Red was selected as the indicator dye solution with an addition of 1 µl from 240 µM to 25 µl of the reaction mixture.

Optimization of the reaction conditions was conducted following the procedures outlined in previous studies [18]. Briefly, temperatures ranging from 57 to 67 °C (in 2 °C intervals) were evaluated to determine the optimal reaction temperature. The optimal reaction time was investigated within the timeframe of 20 to 60 min. Subsequently, a final step involving incubation at 80 °C for a duration of 10 min was implemented for inactivation purposes. Following completion of the reaction, the resulting amplicons were analysed by gel electrophoresis using a 1% agarose gel. Regarding the determination of an appropriate concentration for the neutral red staining solution, consistent amounts were added at concentrations of 180, 210, 240, 270, 300, 330, 360 and 390 µM, respectively. Visual observation of colour changes in the reaction solution facilitated identification of an optimal concentration for neutral red staining. Fig. S2 shows a schematic flow chart of LAMP.

### Specificity and sensitivity of ALSV-LAMP

The specificity of the ALSV-LAMP method was evaluated using four common RNA viruses in ticks, namely ALSV, JMTV, BLTV4 and BJNV. Among them, JMTV is more closely related to ALSV as they both belong to the Flaviviridae family. To assess the sensitivity of ALSV-LAMP, seven gradient dilutions ranging from 0.5 fg μl^−1^ to 0.5 ng µl^−1^ were performed for ALSV cDNA, and eight gradient dilutions ranging from 1.68×10^10 ^to 1.68×10^3^ copies per μl were performed for ALSV-LAMP standards. The results of these evaluations were analysed through 1% agarose gel electrophoresis, neutral red staining and real-time PCR.

## Results

### Establishment of ALSV-LAMP

**Fig. 1.**
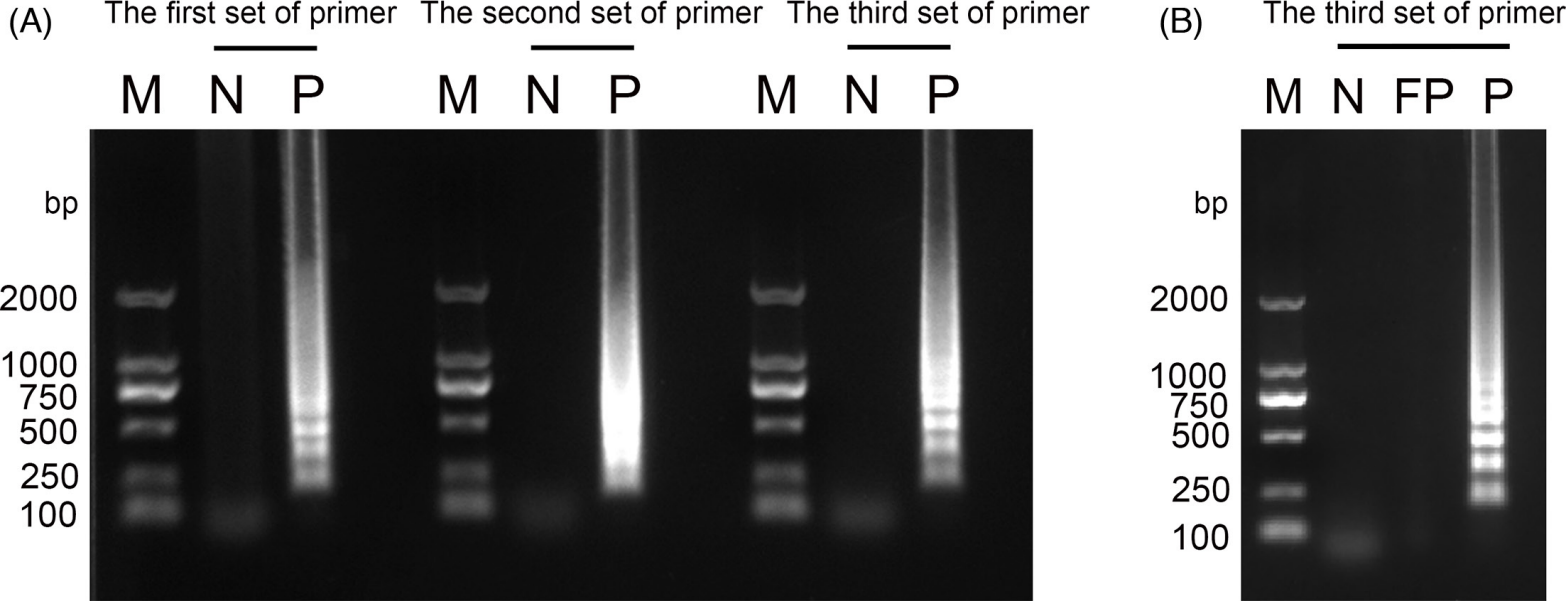
Selection and validation of ALSV-LAMP primers. M, DNA Marker DL2000 (TaKaRa, China); N, no template control; P, LAMP assay using cDNA of ALSV genome as template; FP, LAMP assay using non-ALSV cDNA as template. (A) The effectiveness of LAMP primer set selection was evaluated through 1% gel electrophoresis. Three different sets of candidate LAMP primers were subjected to test. (B) The accuracy of the third primer set was confirmed by false-positive verification. This step validated the credibility and practicality of the chosen third primer set, as evident from the results of 1% gel electrophoresis. The reaction mixture comprised 12.5 µl of 2×Lamp Master Mix, 0.8 µM each of FIP and BIP, 0.2 µM each of F3 and B3, 0.16 U µl^−1^ of DNA Polymerase, 2 µl of template DNA and 5 µl of ddH_2_O.

We designed three sets of LAMP primers, and all three sets were employed in the same reaction system and procedure to analyse both negative and positive samples. The results revealed that the first and third primer sets produced amplified bands for the positive samples. Notably, the third primer set exhibited a distinct trapezoidal distribution characterized by a distinct band pattern (Fig. 1A). The results showed that the banding of the third primer pair was brighter and clearer than that of the first primer pair. At the same time, the band is clearer than the second pair of primers. Consequently, we selected the third primer set for subsequent experiments. Subsequently, we conducted a false-positive assessment using this chosen third primer set. This evaluation demonstrated an absence of discernible amplification bands in both the negative control and negative nucleic acid samples. Conversely, the amplification bands of the positive control were remarkably intense, and the findings collectively emphasize the accuracy and suitability of our selected primers for molecular detection of ALSV (Fig. 1B).

**Fig. 2.**
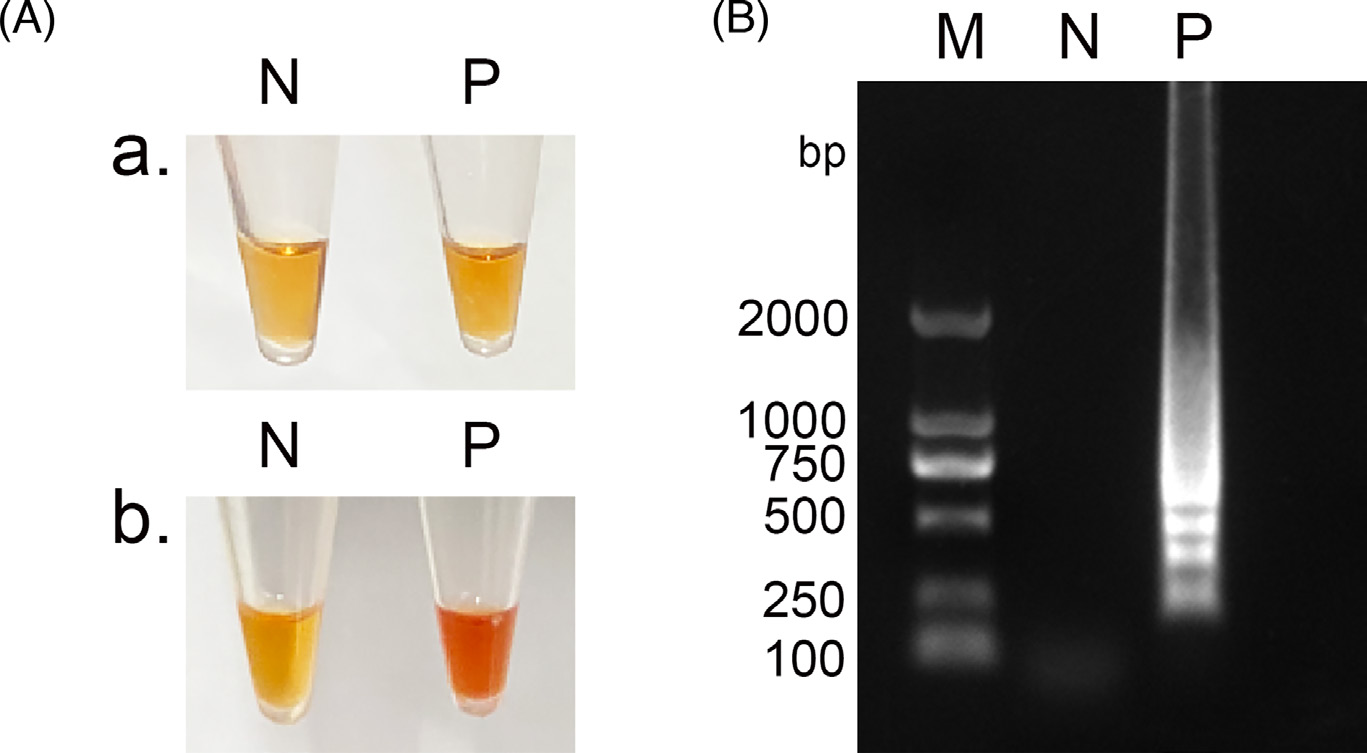
Development of neutral red visualization method. M, DNA Marker DL2000 (TaKaRa, China); N, no template control; P, LAMP assay using cDNA of ALSV genome as template. (A) Neutral red visualization LAMP reaction results a. Prior to the initiation of the neutral red LAMP reaction. b. Post the neutral red LAMP reaction. Visual observation of LAMP products accomplished through the pre-addition of neutral red. A noticeable change in colour from orange to red exclusively occurred in tubes containing ALSV genome, while the negative sample retained its orange hue. (B) Authentication of LAMP assay with neutral red pre-addition through electrophoresis. The product generated using the ALSV genome exhibited a distinct amplified band, congruent with the outcome of neutral red colour change. The reaction mixture composition was as follows: 12.5 µl of 2×Lamp Master Mix, 0.8 µM each of FIP and BIP, 0.2 µM each of F3 and B3, 0.16 U µl^−1^ of DNA Polymerase, 1 µl of neutral red staining solution, 2 µl of template DNA and 4 µl of ddH_2_O.

We developed the LAMP method by utilizing the system and conditions of the 2×Lamp PCR Master Mix (Universal). In preliminary experiments, we incorporated 1 µl of neutral red dye. Upon comparison with the blank control, no discernible change in colour was observed with the naked eye in the negative control reaction solution post-reaction. Conversely, a colour transition from orange to red was observed in the reaction solution (Fig. 2A). To validate the accuracy of neutral red visualization, agarose gel electrophoresis was employed. The results are depicted in Fig. 2B, where distinct ladder-like bands were clearly visible. These bands were further confirmed through sequencing analysis.

### Optimization of ALSV-LAMP

**Fig. 3.**
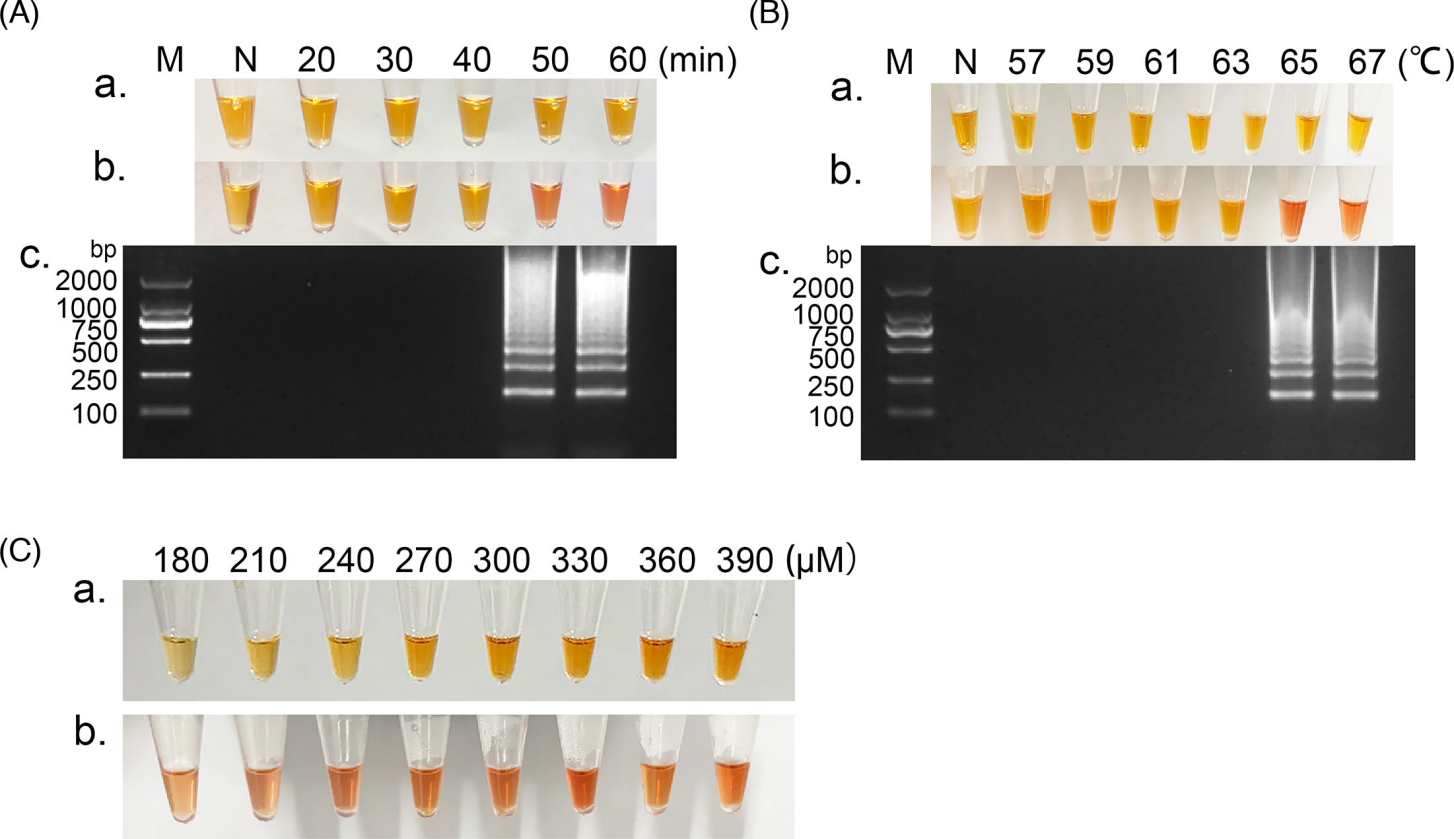
Optimization of ALSV-LAMP reaction. M, DNA Marker DL2000 (TaKaRa, China); N, no template control; a. Neutral red visualization of LAMP before reaction. b. Neutral red visualization after LAMP reaction. c. Agarose gel electrophoresis results. (A) Evaluation of different reaction times in ALSV-LAMP experiments. At 65 °C for 60 min, the negative control exhibited no amplified bands. The graph highlights that amplified bands were evident at 65 °C for 50 min. (B) Examination of various reaction temperatures during ALSV-LAMP. At 65 °C for 50 min, the negative control exhibited no amplified bands. Notably, the graph illustrates that amplified bands appeared at 65 °C and after 50 min of reaction. (C) Refinement of the concentration of neutral red staining solution. The most pronounced colour change within the reaction was achieved at a neutral red concentration of 330 µmol l^−1^. The composition of the reaction mixture was as follows: 12.5 µl of 2×Lamp Master Mix, 0.8 µM each of FIP and BIP, 0.2 µM each of F3 and B3, 0.16 U µl^−1^ of DNA Polymerase, 1 µl of neutral red staining solution, 2 µl of template DNA and 4 µl of ddH_2_O.

The reaction conditions were further optimized, and different incubation times were used for the ALSV-LAMP experiments, including 20, 30, 40, 50 and 60 min. As shown in Fig. 3A, a significant change in the colour of the liquid in the tube can be observed after 50 min of the reaction, from orange for negative results to red for positive results. At the same time, the agarose gel showed clear and distinct trapezoidal bands at 50 and 60 min (Fig. 3A), confirming the results of the reaction. For rapid detection of ALSV, we found that the optimal reaction time for ALSV-LAMP was 50 min.

The incubation temperature of ALSV-LAMP was optimized by a temperature gradient to achieve an optimal reaction time of 50 min. It is noteworthy that clear and bright reaction bands were observed at 65 °C and a clear colour change in the reaction solution was also observed (Fig. 3B). Therefore, we identified 65 °C as the ideal reaction temperature for this study. Subsequently, we employed a single-factor variable experimental visualization of neutral red. At a final concentration of 330 µmol l^−1^, the colour shift exhibited maximum intensity, enabling easy visual detection with the naked eye (Fig. 3C).

### Specificity and sensitivity of ALSV-LAMP

**Fig. 4.**
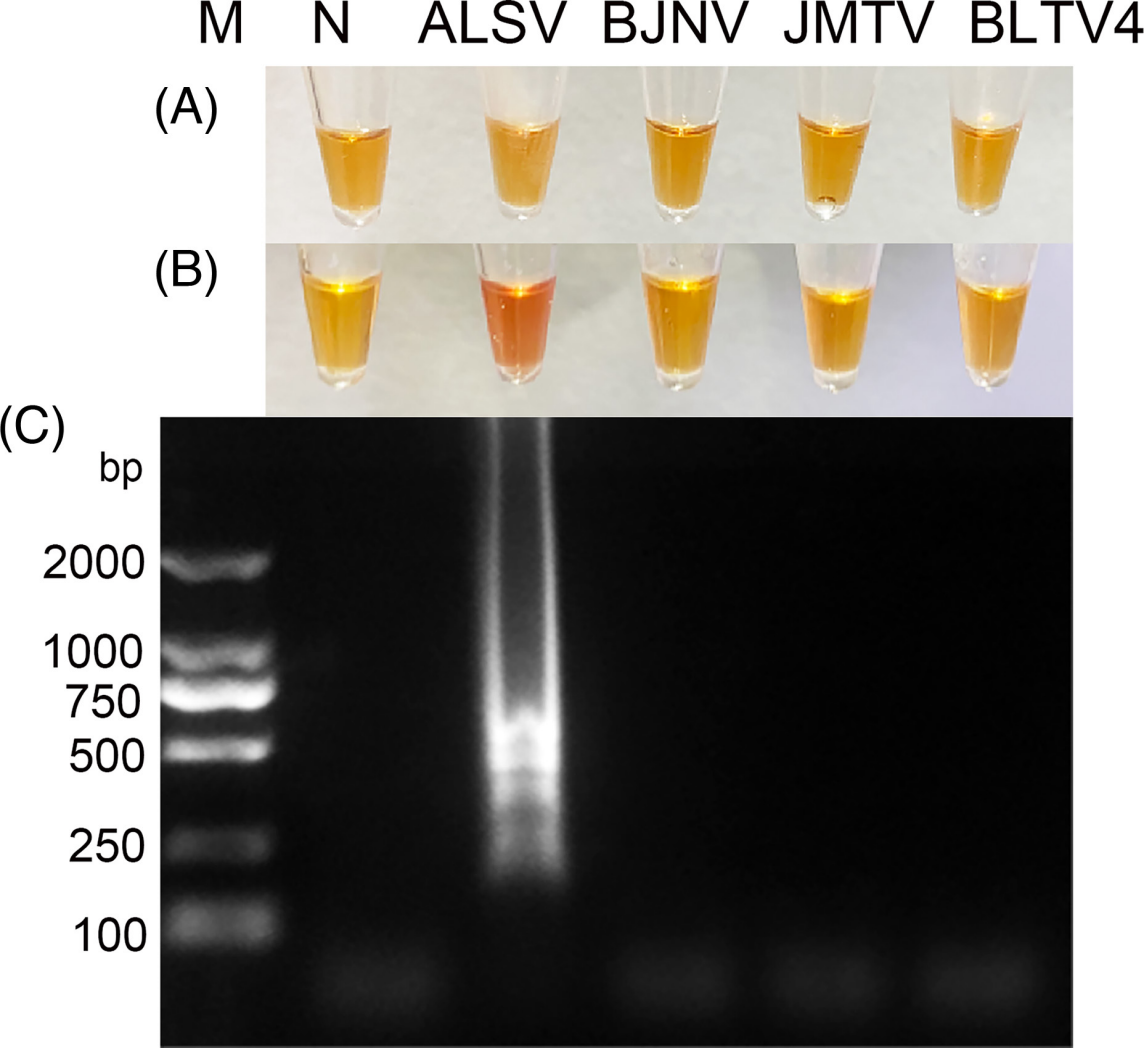
Specificity assessment of ALSV-LAMP assay. M, DNA Marker DL2000 (TaKaRa, China); N, no template control; a. Prior to the initiation of the neutral red LAMP reaction. b. Post the neutral red LAMP reaction. Lane 1, LAMP products using ALSV genome as template; Lane 2, LAMP products using cDNA extracted from JMTV culture medium as template; Lane 3, LAMP products using cDNA extracted from BLTV4 culture medium as template; Lane 4, LAMP products using cDNA extracted from the tick samples as template. (A, B) Result of colourimetric analysis using neutral red staining solution. The reaction solution with the ALSV template exhibited a change in colour to red. (C) Agarose gel electrophoresis result. The reaction solution with the ALSV template showed an amplified band, consistent with the colour change due to neutral red. The composition of the reaction mixture was as follows: 12.5 µl of 2×Lamp Master Mix, 0.8 µM each of FIP and BIP, 0.2 µM each of F3 and B3, 0.16 U µl^−1^ of DNA polymerase, 1 µl of neutral red staining solution, 2 µl of template DNA and 4 µl of ddH_2_O.

We performed an assessment to evaluate the analytical specificity of ALSV-LAMP using JMTV, BLTV4 and BJNV. Encouragingly, no instances of cross-reaction were observed with these viruses. Conversely, a positive result is evident in the ALSV-positive sample, highlighting the enhanced specificity of ALSV-LAMP (Fig. 4).

**Fig. 5.**
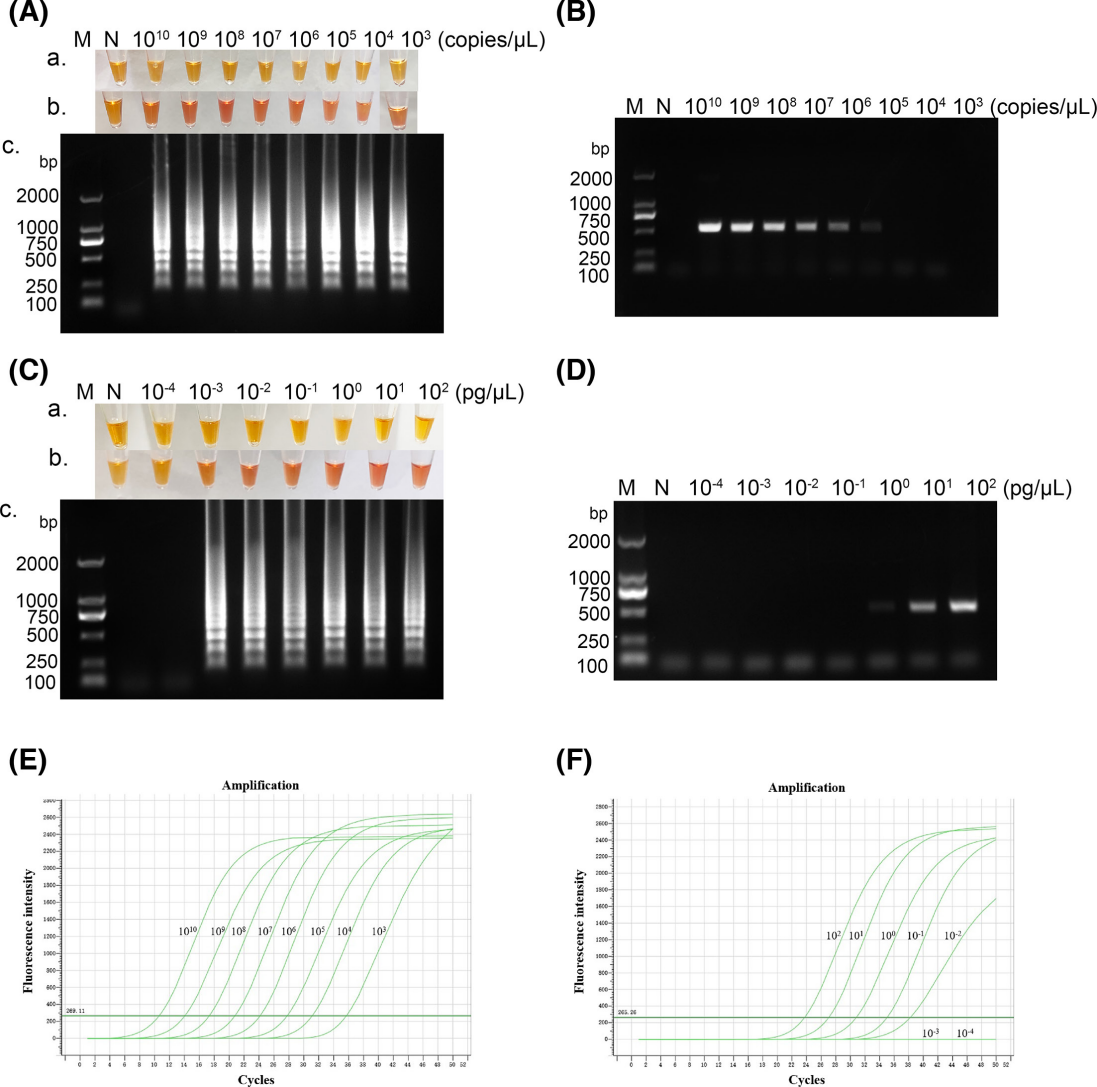
Sensitivity analysis of LAMP, PCR and real-time PCR assays. M, DNA Marker DL2000 (TaKaRa, China); N, no template control; a. Neutral red visualization of LAMP before reaction. b. Neutral red visualization after LAMP reaction. c. Agarose gel electrophoresis results. A 10-fold serial dilution (from 1.68×10^10^ to 1.68×10^3^ copies per μl) of the ALSV-LAMP standard was used as a sample for the sensitivity testing. The evaluation of minimum detection limit was executed using serial dilutions of ALSV-cDNA, spanning from 5×10^2^ to 5×10^−4^ pg µl^−1^. (A) Sensitivity testing of the ALSV-LAMP assay. (B) Sensitivity testing of the ALSV-PCR assay. (C) Minimum detection limit of LAMP. (D) Minimum detection limitation of PCR. (E) Sensitivity testing of the ALSV real-time PCR assay. (F) Minimum detection limitation of real-time PCR. The assay detected down to 0.005 pg µl^−1^ per reaction of ALSV-cDNA for LAMP, and 5 pg µl^−1^ for PCR and real-time PCR, which showed that the sensitivity of LAMP method was 1,000 times that of PCR and real-time PCR. The composition of the reaction mixture was as follows: 12.5 µl of 2×Lamp Master Mix, 0.8 µM each of FIP and BIP, 0.2 µM each of F3 and B3, 0.16 U µl^−1^ of DNA polymerase, 2 µl of template DNA and 5 µl of ddH_2_O.

In the sensitivity assessment, a 10-fold gradient dilution was employed for LAMP, PCR and real-time PCR standards. Interestingly, LAMP exhibited a distinctive amplified band at a concentration of 1.68×10^3^ copies per μl (Fig. 5A), whereas PCR only detected a faint target band at 1.68×10^5^ copies per μl (Fig. 5B). The real-time PCR has exceeded 35 cycles at 1.68×10^3^ copies per μl, which can be determined as negative (Fig. 5E). Additionally, ALSV-LAMP experiments were conducted to determine the minimum detection limit. Seven RNA concentrations were examined: 0.5 fg μl^−1^, 5 fg μl^−1^, 50 fg μl^−1^, 0.5 pg µl^−1^, 5 pg µl^−1^, 50 pg µl^−1^ and 0.5 ng µl^−1^. The results, as depicted in Fig. 5C, demonstrated that LAMP successfully detected a positive band at an RNA concentration of 5 fg μl^−1^. In contrast, PCR only detected a positive band starting from an RNA concentration of 5 pg µl^−1^ (Fig. 5D), and real-time PCR exceeded 35 cycles at 0.5 pg µl^−1^ (Fig. 5F). Therefore, the sensitivity of the LAMP method was 1,000 times higher than that of PCR and real-time PCR. The minimum detection limit of LAMP for ALSV cDNA was established at 5 fg μl^−1^, reaffirming the exceptional sensitivity of ALSV-LAMP in detecting ALSV.

## Discussion

ALSV is an emerging tick-borne zoonotic virus, and the current PCR assays are not efficient for rapid clinical diagnosis of ALSV infection. In this study, we developed a LAMP assay for the detection of ALSV infection, with an optimal temperature set at 65 °C. The LAMP assay exhibited a minimum detection concentration of 0.005 pg µl^−1^, which was 1,000-fold higher than that achieved by PCR. Compared with traditional assays, the ALSV-LAMP assay presents significant advantages such as thermostability and colour development, rendering it suitable for field applications. Conversely, assays like PCR and real-time PCR necessitate precise thermal cycling during DNA amplification and intricate procedures. Additionally, the cost of detection for PCR and real-time PCR is considerably higher compared with that of LAMP.

In this investigation, following the ladder temperature model, we observed a robust amplification efficiency with the initial LAMP reaction occurring at 65 ℃ for 50 min. Consequently, we hypothesized that the optimal temperature for the LAMP reaction in our study was 65 ℃.

Initially, positive results were identified using agarose gel electrophoresis to detect trapezoidal bands. However, it became apparent that cross-contamination posted an inevitable challenge during this process. In 2001, Mori reported the generation of magnesium pyrophosphate as a by-product of the LAMP reaction, resulting in a transition from clarity to turbidity within the reaction tube [27]. Although turbidity-based observation has been employed, the associated costs of utilizing turbidimeters for analysis have limitations.

To overcome these challenges, we opted for the dye method due to its superior convenience and accuracy compared to the turbidimetric approach. By utilizing hydroxynaphthol blue dye, which releases magnesium ions through the formation of magnesium pyrophosphate during the LAMP reaction, a discernible colour change from violet to sky blue was observed. However, this transition was not sufficiently pronounced and could potentially lead to misinterpretation [28,29]. Similarly, the utilization of SYBR Green showed significant disparities between positive and negative reactions. However, the incorporating SYBR Green I dye presented a challenge as it had to be added at the conclusion of the reaction in order to circumvent its inhibitory effect, thereby increasing the likelihood of experimental contamination due to tube opening [30].

In our study, we strategically selected a neutral red stain as the indicator of reaction outcomes due to its ability to undergo colour alteration in response to fluctuating pH levels in the reaction solution. The positive reaction exhibited a transition from orange to red, while the hue remained unchanged in the negative reaction tube. By incorporating neutral red dye into our experimental setup, we significantly expedited the time efficiency.

Consequently, the developed ALSV-LAMP assay provides a more pragmatic approach for routine applications compared with traditional PCR and real-time PCR assays. ALSV showed relatedness to JMTV than other Jingmen viruses [1,23, 31]. The genomes of ALSV and JMTV consist of four segments, two of which share similarities with the NS3 and NS5 proteins found in non-segmented RNA viruses belonging to the genus *Flavivirus* [9]. In this study, several primer pairs were designed and screened based on conserved regions within the S1 segment. Subsequently, false-positive validation was conducted to ensure primer accuracy and applicability. The results demonstrated that the LAMP method exhibited no cross-reactivity within JMTV, BJNV or BLTV4 tick-borne viruses, confirming its specificity for ALSV detection. Furthermore, the detection limit of the LAMP assay for ALSV is much lower, approximately 1,000 times lower than that of PCR and real-time PCR.

Although the LAMP technique is widely utilized, it is imperative to acknowledge its limitations. LAMP exhibits a high degree of sensitivity to aerosol contamination, thereby resulting in false-positive outcomes. The design of LAMP primers poses challenges as it necessitates highly conserved target sequences and primer lengths for ensuring specific amplification. Moreover, the exceptional specificity of the LAMP assay restricts its applicability in studying novel genes with limited information or target genes of unknown structures. In conclusion, the recently developed ALSV-LAMP assay represents a simple, rapid, specific, highly sensitive and visually detectable method that holds great potential as an alternative for the clinical diagnosis of ALSV.

## Supplementary material

10.1099/jgv.0.002094 Uncited Supplementary Material 1.

## Abbreviations

ALSV: Alongshan virus
BIP: backward inner primer
BLTV4: Bole tick virus 4
Bst: *Bacillus stearothermophilus*
FIP: forward inner primer
HIV: human immunodeficiency virus
JMTV: Jingmen tick virus
LAMP: loop-mediated isothermal amplification
RT-PCR: reverse transcription PCR
