# Supplementary material for: Development of a loop-mediated isothermal amplification assay for the rapid detection of Alongshan virus
Source: J Gen Virol. 2025 May 8;106(5):002094. doi: 10.1099/jgv.0.002094 (PMC12062536; doi:10.1099/jgv.0.002094)
Supplement: Uncited Supplementary Material 1. [file jgv-106-02094-s001.pdf]

Supplement 1: Schematic representation of position and sequence of primer sets within the nucleotide sequence of the genome S1 segmentsequence of ALSV strain NE-TH4 (GenBank Accession no. ON408067.1) used for LAMP assay.

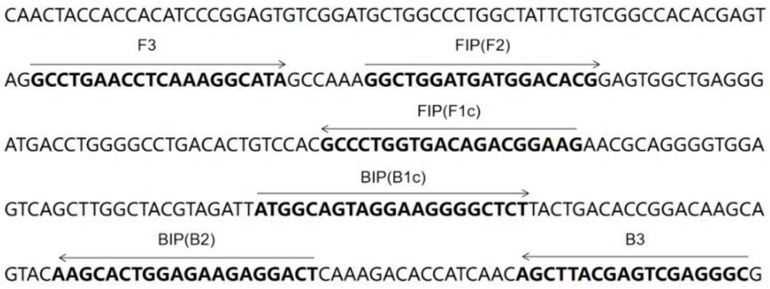

Supplement 2:Schematic flow chart of Loop-Mediated Isothermal Amplification

The principle of LAMP primers

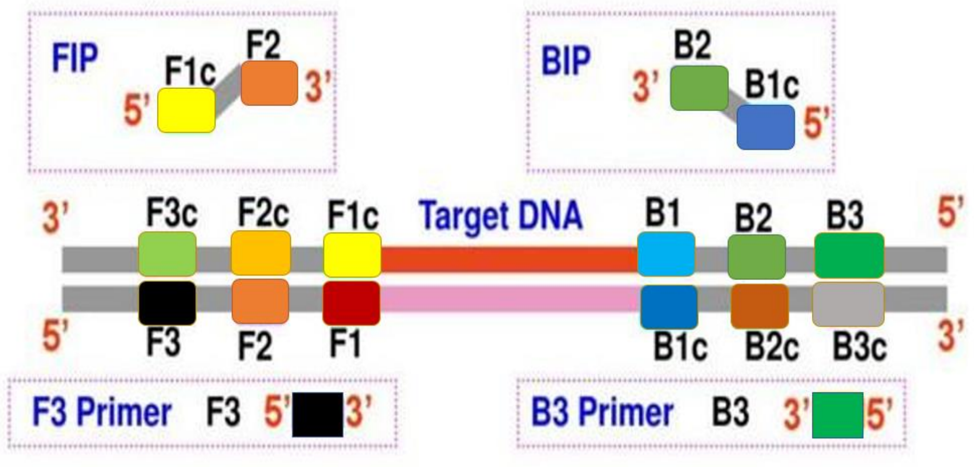

The formation process of dumbbell formwork structure

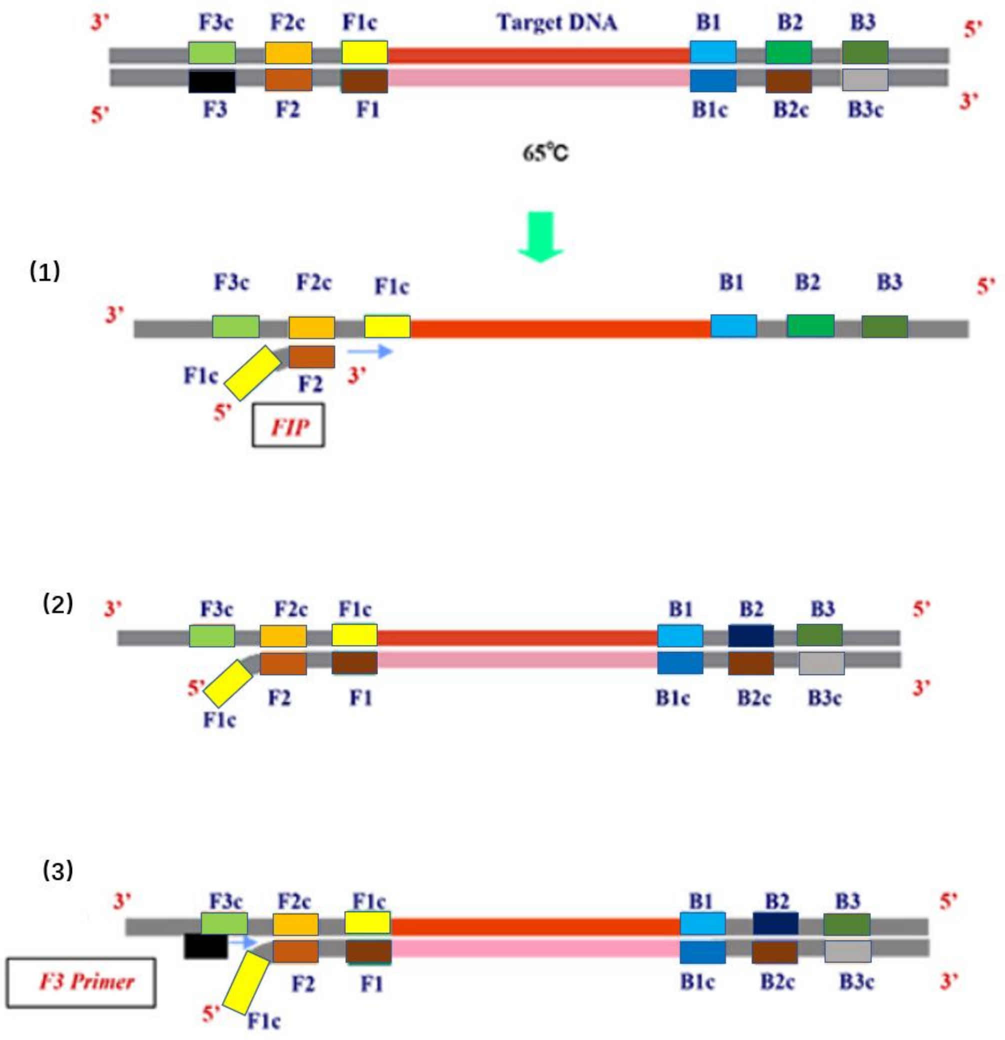

(4)

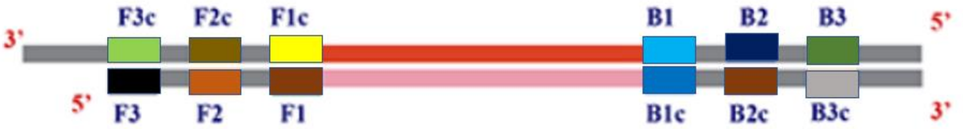

(5)

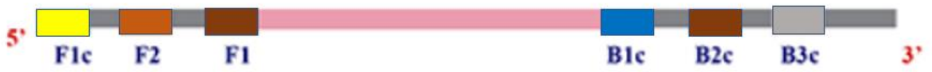

(6)

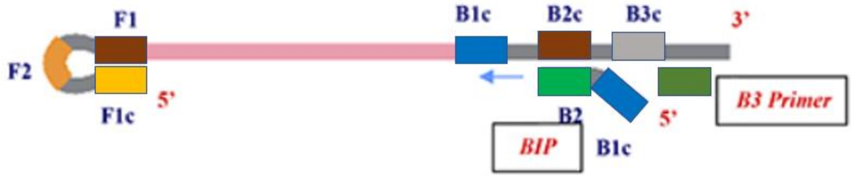

(7)

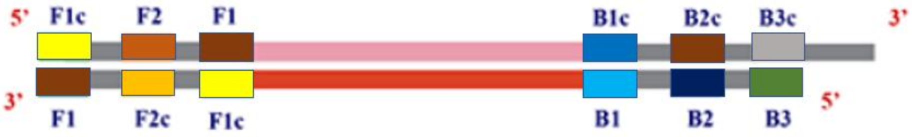

(8)

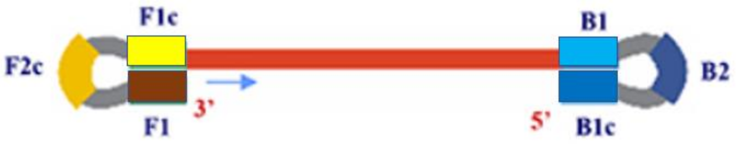

The phase of circle amplification

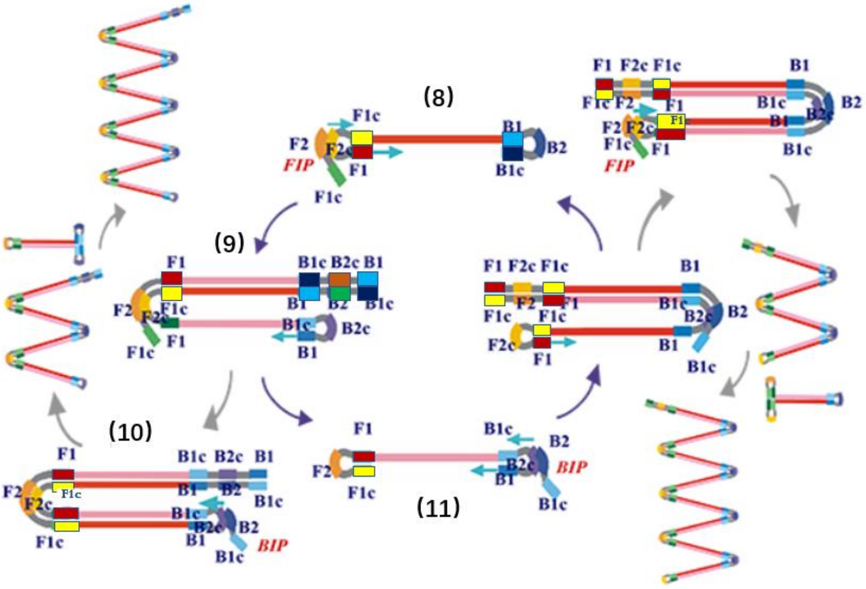
(8)
(9)
(10)
(11)
FIP
BIP
F1
F2
F1c
F2c
B1
B2
B1c
B2c
